# Emergence and Spread of Carbapenem-Resistant and Aminoglycoside-Panresistant *Enterobacter cloacae* Complex Isolates Coproducing NDM-Type Metallo-β-Lactamase and 16S rRNA Methylase in Myanmar

**DOI:** 10.1128/mSphere.00054-20

**Published:** 2020-03-11

**Authors:** Satoshi Oshiro, Tatsuya Tada, Shin Watanabe, Mari Tohya, Tomomi Hishinuma, Hiroki Uchida, Kyoko Kuwahara-Arai, San Mya, Khin Nyein Zan, Teruo Kirikae, Htay Htay Tin

**Affiliations:** a Department of Microbiology, Juntendo University School of Medicine, Tokyo, Japan; b Department of Microbiome Research, Juntendo University School of Medicine, Tokyo, Japan; c National Health Laboratory, Yangon, Myanmar; Antimicrobial Development Specialists, LLC

**Keywords:** NDM-type metallo-β-lactamase, 16S rRNA methylase, *Enterobacter cloacae* complex

## Abstract

Surveillance of 10 hospitals and a regional public health laboratory in Myanmar identified 31 isolates of carbapenem-resistant Enterobacter cloacae complex harboring *bla*_NDM-type_. Of these isolates, 19 were highly resistant to aminoglycosides and harbored one or more genes encoding 16S rRNA methylases, including *armA*, *rmtB*, *rmtC*, and/or *rmtE*. Of the 19 isolates, 16 were Enterobacter xiangfangensis ST200, with *armA* on the chromosome and a plasmid harboring *bla*_NDM-1_ and *rmtC*, indicating that these isolates were clonally disseminated nationwide in Myanmar.

**IMPORTANCE** The emergence of multidrug-resistant E. cloacae complex has become a public health threat worldwide. *E. xiangfangensis* is a recently classified species belonging to E. cloacae complex. Here, we report a clonal dissemination of multidrug-resistant *E. xiangfangensis* ST200 producing two types of New Delhi metallo-β-lactamase (NDM-type MBL), NDM-1 and -4, and three types of 16S rRNA methylases, ArmA, RmtC, and RmtE, in hospitals in Myanmar. The observation of these multidrug-resistant *E. xiangfangensis* ST200 isolates stresses the urgency to continue molecular epidemiological surveillance of these pathogens in Myanmar and in South Asian countries.

## Abstract (precis)

The emergence of multidrug-resistant E. cloacae complex has become a public health threat worldwide. *E. xiangfangensis* is a recently classified species belonging to E. cloacae complex. Here, we report a clonal dissemination of multidrug-resistant *E. xiangfangensis* ST200 producing two types of New Delhi metallo-β-lactamase (NDM-type MBL), NDM-1 and -4, and three types of 16S rRNA methylases, ArmA, RmtC, and RmtE, in hospitals in Myanmar. The observation of these multidrug-resistant *E. xiangfangensis* ST200 isolates stresses the urgency to continue molecular epidemiological surveillance of these pathogens in Myanmar and in South Asian countries.

## INTRODUCTION

Metallo-β-lactamases (MBLs) are the most important enzymes contributing to carbapenem resistance (1). New Delhi MBL (NDM-type MBL) was initially reported in Klebsiella pneumoniae and Escherichia coli isolates from a patient who had traveled from India to Sweden in 2008 (2). Since then, NDM-type MBL-producing Gram-negative pathogens have been isolated from patients worldwide (3).

Acquired 16S rRNA methylases are responsible for high-level resistance to various aminoglycosides in Gram-negative pathogens, including *Enterobacteriaceae* (4). The 16S rRNA methylases ArmA and RmtA were first identified in isolates of K. pneumoniae and Pseudomonas aeruginosa, respectively (3). Subsequently, 10 classes of 16S rRNA methylases, including ArmA, RmtB, RmtC, and RmtE, have been detected in clinical isolates of Gram-negative bacteria (3). The geographic distribution of these isolates has been shown to depend on classes of 16S methylase. For example, ArmA and RmtB producers have been detected worldwide, RmtC producers in India and the United Kingdom and RmtE producers in the United States and China (3, 5).

This report describes the spread of multidrug-resistant Enterobacter cloacae complex isolates producing various NDM-type MBLs (NDM-1, -4, -5, and -7) and 16S rRNA methylases (ArmA, RmtB, RmtC, and RmtE) throughout hospitals in Myanmar.

## RESULTS AND DISCUSSION

### Multidrug-resistant E. cloacae complex.

Ten hospitals and a regional public health laboratory in Myanmar were surveilled for carbapenem-resistant Gram-negative pathogens between December 2015 and May 2017. Carbapenem-resistant isolates were screened using the Vitek 2 system (bioMérieux, Marcy l’Etoile, France), resulting in the detection of 31 isolates of E. cloacae complex resistant to imipenem and/or meropenem from patients at the 10 hospitals and the regional public health laboratory in five regions of Myanmar. These participating facilities were requested to submit carbapenem-resistant clinical isolates; therefore, it is unknown how many isolates were screened in this surveillance. The 31 carbapenem-resistant isolates included 20 from six hospitals in the Yangon Region, four from a hospital and the regional public health laboratory in the Mandalay Region, one from a hospital in the Magway Region, four from a hospital in Mon State and two from a hospital in Naypyidaw Union Territory (see Table S1 and Fig. S1 in the supplemental material). Of the 31 isolates, 15 were obtained from urine samples, six from wound swabs, four from blood, three from pus, two from respiratory tracts, and one from peritoneal dialysis fluid (Table S1). All these isolates were positive by CIMTrisII (Kohjin Bio, Saitama, Japan), an improved carbapenem inactivation method for the detection of carbapenemase production (6), and all of them were positive on an immunochromatography assay for NDM-type MBL (7). Antimicrobial susceptibility of these isolates to various antibiotics was tested by the microdilution method according to guidelines of the Clinical and Laboratory Standards Institute (8) and the U.S. Food and Drug Administration (9) (Table 1). All the 31 isolates were resistant to carbapenems, including 28 resistant to both imipenem and meropenem and three resistant to either imipenem or meropenem; 19 isolates (61%) were resistant to amikacin; 25 isolates (81%) were resistant to ciprofloxacin; two were resistant to colistin; and 19 were extremely resistant to amikacin and arbekacin with MICs of ≥1,024 μg/ml (Table 1 and Table S1). No isolates were resistant to tigecycline (Table 1).

**TABLE 1:** MIC_50_ and MIC_90_ values and percentages of resistance among E. cloacae complex isolates^*a*^

Antibiotic	Breakpoint for resistance (μg/ml)	% resistance	MIC (μg/ml)		
			Range	MIC_50_	MIC_90_
Amikacin	≥64	61	0.5 to ≥1,024	≥1,024	≥1,024
Arbekacin			<0.25 to ≥1,024	≥1,024	≥1,024
Aztreonam	≥14	81	0.5 to ≥1,024	256	≥1,024
Ceftazidime	≥64	100	≥1,024	≥1,024	≥1,024
Ciprofloxacin	≥4	81	0.25 to ≥1,024	128	256
Colistin	>2	6	0.0016 to ≥16	0.125	0.5
Imipenem	≥4	94	2 to 256	16	64
Meropenem	≥4	97	2 to 256	32	128
Tigecycline	≥8	0	0.125 to 2	1	2

a Breakpoints for microbial resistance were determined according to the guidelines of the Clinical and Laboratory Standards Institute for amikacin, aztreonam, ceftazidime, ciprofloxacin, imipenem, and meropenem; of the European Committee on Antimicrobial Susceptibility Testing for colistin; and of the U.S. Food and Drug Administration for tigecycline.

10.1128/mSphere.00054-20.1 FIG S1 Locations of 10 hospitals and a regional public health laboratory in Myanmar from which isolates of carbapenem-resistant E. cloacae complex were obtained and the numbers of isolates per facility. Download FIG S1, PDF file, 0.03 MB. Copyright © 2020 Oshiro et al. 2020 Oshiro et al. This content is distributed under the terms of the Creative Commons Attribution 4.0 International license.

10.1128/mSphere.00054-20.4 TABLE S1 Isolate information. Download Table S1, XLSX file, 0.02 MB. Copyright © 2020 Oshiro et al. 2020 Oshiro et al. This content is distributed under the terms of the Creative Commons Attribution 4.0 International license.

### Drug resistance genes from E. cloacae complex.

All 31 isolates of E. cloacae complex harbored one of four genes encoding NDM-type MBLs, including *bla*_NDM-1_ (25 isolates), *bla*_NDM-4_ (4 isolates), *bla*_NDM-5_ (one isolate), and *bla*_NDM-7_ (one isolate). Of these isolates, 19 harbored one, two, or three genes encoding 16S rRNA methylases, including *armA*, *rmtB*, *rmtC*, and/or *rmtE.* Specifically, 11 harbored *armA* and *rmtC*, and five harbored *armA*, *rmtC*, and *rmtE* (Table 2 and Table S1). Most of the 31 isolates harbored the plasmid-mediated quinolone resistance genes *aac(6′)-lb-cr* (*n* = 26) (these genes of the 26 isolates were confirmed in GenBank [accession no. DQ303918 and EF636461]) and *qnr* (*n* = 29) (10) and had amino acid substitutions in DNA gyrase (Ser/Thr83 and/or Asp87 in GyrA) (*n* = 25) and topoisomerase IV (Ser80 in ParC) (*n* = 16) (Table 2 and Table S1). The two colistin-resistant isolates had amino acid substitutions in PmrB (Gly275Asp and Phe112Tyr/Asp274Glu/Gln294Glu, respectively), which are associated with greater colistin resistance than in type strains (Table S1).

**TABLE 2:** MLSTs and drug resistance genes of the 31 E. cloacae complex isolates

MLST	No. of isolates	Gene(s) encoding:	
		Carbapenemase and β-lactamase	16S rRNA methylase and aminoglycoside acetyltransferase
ST66	1	*bla*_NDM-1_, *bla*_ACT-7_, *bla*_TEM-1B_	*aadA1*, *aph(3″)-lb*, *aph(3′)-Vl*, *aph(6)-ld*
ST90	1	*bla*_NDM-1_, *bla*_ACT-15_, *bla*_CTX-M-15_, *bla*_DHA-1_	*armA*, *aac(6')-lb3*, *aadA1b*, *aac(6')-lb-cr*
ST114	2	*bla*_NDM-1_, *bla*_ACT-16_, *bla*_CTX-M-15_ (1/2), *bla*_DHA-1_	*aac(6')-lb3*, *aac(6')-lb-cr*
ST171	2	*bla*_NDM-1_ (1/2), *bla*_NDM-4_ (1/2), *bla*_ACT-7_, *bla*_CTX-M-15_ (1/2), *bla*_OXA-1_ (1/2), *bla*_TEM-1B_ (1/2)	*aac(3)-lla* (1/2), *aadA1*, *aadA2* (1/2), *aph(3')-la* (1/2), *aph(3”)-lb* (1/2), *aph(6)-ld* (1/2), *aac(6')-lb-cr* (1/2)
ST182	1	*bla*_NDM-1_, *bla*_ACT-16_, *bla*_TEM-1B_	*aadA1*, *aph(3”)-lb*, *aph(6)-ld*
ST200	18	*bla*_NDM-1_ (16/18), *bla*_NDM-4_ (2/18), *bla*_CTX-M-15_, *bla*_OXA-1_ (1/18), *bla*_TEM-1B_	*armA* (16/18), *rmtC* (16/18), *rmtE* (5/18), *aac(3)-lld* (16/18), *aac(6')-lb3* (16/18), *aadA2* (16/18), *aph(3')-Vl* (14/18), *aac(3)-lla* (2/18), *aadA1* (2/18), *aph(3”)-lb* (2/18), *aph(6)-ld* (2/18), *aac(6')-lb-cr*
ST312	1	*bla*_NDM-4_, *bla*_ACT-7_, *bla*_CTX-M-15_, *bla*_OXA-1_, *bla*_OXA-10_, *bla*_TEM-1B_, *bla*_VEB-1_	*aac(3)-lla*, *aadA1*, *aadA2*, *ant(2”)-la*, *aph(3”)-lb*, *aph(3')-la*, *aph(6)-ld*, *aac(6')-lb-cr*
ST513	1	*bla*_NDM-1_, *bla*_CTX-M-15_, *bla*_DHA-1_, *bla*_OXA-1_, *bla*_TEM-1B_	*armA*, *aac(3)-lla*, *aac(6')-lb3*, *aadA1*, *aph(3”)-lb*, *aph(6)-ld*, *aac(6')-lb-cr*
ST916	1	*bla*_NDM-7_, *bla*_ACT-7_, *bla*_CTX-M-15_, *bla*_DHA-1_, *bla*_OXA-1_, *bla*_TEM-1B_	*aac(6')-llc*, *aph(3”)-lb*, *aph(6)-ld*, *aac(6')-lb-cr*
ST1053	1	*bla*_NDM-1_, *bla*_ACT-16_, *bla*_CTX-M-15_, *bla*_DHA-1_, *bla*_OXA-1_, *bla*_TEM-1B_	*aac(3)-lla*, *aac(6')-lb3*, *aadA1*, *aph(3”)-lb*, *aph(6)-ld*, *aac(6')-lb-cr*
ND^*a*^	1	*bla*_NDM-5_, *bla*_MIR-5_, *bla*_TEM-1B_	*rmtB*, *aadA2*, *aph(3”)-lb*, *aph(6)-ld*
ND^*b*^	1	*bla*_NDM-1_, *bla*_ACT-7_, *bla*_CTX-M-15_, *bla*_DHA-1_, *bla*_OXA-1_, *bla*_TEM-1B_	*aac(3)-lla*, *aac(6')-lb3*, *aadA1*, *aph(3”)-lb*, *aph(6)-ld*, *aac(6')-lb-cr*

a ND, not determined. An isolate of *E. nimipressuralis* did not harbor *lueS*, a housekeeping gene used to determine E. cloacae MLST.

b Not determined. An isolate of *E. xiangfangensis* did not harbor *pyrG*, a housekeeping gene used to determine E. cloacae MLST.

### Drug susceptibility phenotype and drug resistance genotype.

As shown by drug susceptibility phenotypes and drug resistance genotypes of individual isolates in Table S1, all the 31 isolates harbored *bla*_NDM-type_, with 28 being resistant to both imipenem and meropenem and the remaining 3 resistant to either imipenem or meropenem. Nineteen isolates were extremely resistant to aminoglycosides and harbored 16S rRNA methylase-encoding genes, whereas the remaining 12 did not. Twenty-five isolates were resistant to ciprofloxacin, harbored plasmids bearing the quinolone resistance genes *aac(6′)-lb-cr* and/or *qnr*, and had amino acid substitutions in GyrA and/or ParC. The remaining six were susceptible to ciprofloxacin, although they harbored *aac(6′)-lb-cr* and/or *qnr* and did not have any mutations in *gyrA* and *parC*. Of 2 colistin-resistant isolates with amino acid substitutions in PmrB, an isolate with one amino acid substitution (Gly275Asp) showed a higher MIC of colistin (≥256 μg/ml) than that for another isolate with three amino acid substitutions (Phe112Tyr/Asp274Glu/Gln294Glu) (16 μg/ml), indicating that the Gly275Asp substitution plays a critical role in high colistin resistance.

### Phylogenetic analysis.

**FIG 1:**
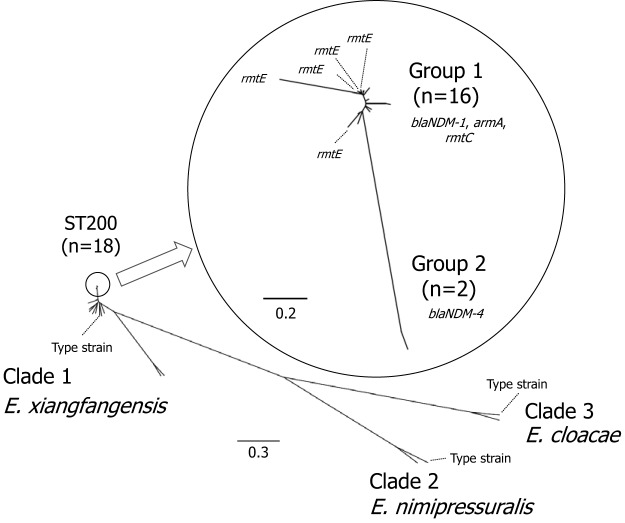
Molecular phylogeny of the E. cloacae complex. A phylogenetic tree involving the 31 isolates and three type strains (*E. xiangfangensis* LMG27195, E. cloacae ATCC 13047, and *E. nimipressuralis* DSM18955) was constructed using kSNP3 (14).

Multilocus sequence typing (MLST) revealed that 29 of all the 31 isolates belonged to one of 10 sequence types (STs) and the STs of the remaining 2 isolates were not determined. Of the 29 isolates, 18 belonged to ST200 (Table 2). Average nucleotide identity (ANI) calculation and digital DNA-DNA hybridization (dDDH) revealed that, of the 31 isolates, 29 were Enterobacter xiangfangensis, one was E. cloacae, and one was Enterobacter nimipressuralis (Table S2). Phylogenetic analysis of the 31 E. cloacae complex isolates revealed three clades, in good agreement with the three species of E. cloacae complex, with 29 isolates of *E. xiangfangensis* belonging to clade 1 and one isolate each of E. cloacae and *E. nimipressuralis* belonging to the other two clades. Of the 29 isolates belonging to clade 1, 18 belonged to ST200, with 16 subclustered in the phylogenetic tree to group 1 and two clustered to group 2 (Fig. 1). All the isolates belonging to group 1 harbored *bla*_NDM-1_, *armA*, and *rmtC*, with five of these also harboring *rmtE*. The two isolates belonging to group 2 harbored *bla*_NDM-4_ but did not harbor any genes encoding a 16S rRNA methylase.

10.1128/mSphere.00054-20.5 TABLE S2 Results of ANI calculation and dDDH for 31 E. cloacae complex isolates. *^a^*E. cloacae (ATCC 13047), *E. asburiae* (ATCC 35953), *E. hormaechei* (ATCC 49162), *E. kobei* (DSM13645), *E. ludwigii* (EN-119), *E. nimipressuralis* (DSM18955), and *E. xiangfangensis* (LMG27195) were used in this analysis as type strains. Download Table S2, XLSX file, 0.01 MB. Copyright © 2020 Oshiro et al. 2020 Oshiro et al. This content is distributed under the terms of the Creative Commons Attribution 4.0 International license.

### Pulsed-field gel electrophoresis and Southern hybridization.

Pulsed-field gel electrophoresis and Southern hybridization were performed using 18 isolates belonging to ST200. Of them, 16 harbored *bla*_NDM-1_ and *rmtC* in a plasmid and *armA* on a chromosome and two harbored *bla*_NDM-4_ in a plasmid (Fig. S2). Further, of the 16 isolates, *bla*_NDM-1_ and *rmtC* were detected in same-size plasmids and three isolates harbored *rmtE* in a plasmid (Fig. S2).

10.1128/mSphere.00054-20.2 FIG S2 Pulsed-field gel electrophoresis and Southern hybridization. Probes for *bla*_NDM-type_, *armA*, *rmtC*, and *rmtE* were used to detect *bla*_NDM-type_, *armA*, *rmtC*, and *rmtE*, respectively. (A) Locations of *bla*_NDM-type_, *rmtC*, and *rmtE* on plasmid DNA. (B) Location of *armA* on chromosomal DNA. Lanes: 1, MY10; 2, MY87; 3, MY92; 4, MY146; 5, MY161; 6, MY204-1; 7, MY262; 8, MY309; 9, MY454-2; 10, MY458; 11, MY460; 12, MY464; 13, MY506; 14, MY633; 15, MY666; 16, MY811; 17, MY82; 18, MY758. Download FIG S2, PDF file, 0.1 MB. Copyright © 2020 Oshiro et al. 2020 Oshiro et al. This content is distributed under the terms of the Creative Commons Attribution 4.0 International license.

### Comparative analysis of *E. xiangfangensis* ST200.

The whole-genome sequences of three isolates of *E. xiangfangensis* harboring *armA*, *rmtC*, and *rmtE* were determined using MinION and MiSeq. Reads generated by these sequencers were assembled, yielding the complete sequences of the chromosomes and plasmids of these three isolates (Table S3). The chromosomes of the three *E. xiangfangensis* isolates were similar in size (5.2 Mbp). The three *E. xiangfangensis* isolates had three or four plasmids, of which a 57,085-bp plasmid harbored both *bla*_NDM-1_ and *rmtC* in three isolates (MY146, MY458, and MY460). The sequence of the 57,085-bp plasmid had 99% similarity with that of the 57,089-bp plasmid pM308-NDM1 (accession no. AP018832), which had been isolated from *E. xiangfangensis* obtained in Myanmar (11). The three isolates also had a plasmid harboring an identical genetic environment of *rmtE*, but the sizes were different among them, 118,668 bp (accession no. LC511996), 119,259 bp (accession no. LC511995), and 345,906 bp (accession no. LC511997), respectively (Table S3). The sequence of the 118,668-bp plasmid had 99% similarity with that of the 119,259-bp plasmid (Fig. S3). The 345,906-bp plasmid had a structure similar to that of the 118,668-bp and 119,259-bp plasmids (Fig. S3). The other structure of the 345,906-bp plasmid was similar to that of pSJO-60984 (accession no. CP025277) (Fig. S3). Collectively, these results of MinION and MiSeq analyses of the three isolates were consistent with the results of Southern hybridization of the 16 isolates.

10.1128/mSphere.00054-20.3 FIG S3 Identical genetic environment of *rmtE*. Comparison of three plasmids harboring *rmtE* with pSJO-60984 (accession no. CP025277). Download FIG S3, PDF file, 0.2 MB. Copyright © 2020 Oshiro et al. 2020 Oshiro et al. This content is distributed under the terms of the Creative Commons Attribution 4.0 International license.

10.1128/mSphere.00054-20.6 TABLE S3 Drug resistance genes in chromosome and plasmid in 3 isolates belonging to ST200. Download Table S3, XLSX file, 0.01 MB. Copyright © 2020 Oshiro et al. 2020 Oshiro et al. This content is distributed under the terms of the Creative Commons Attribution 4.0 International license.

Carbapenem-resistant *E. xiangfangensis* clonal complex 200 (CC200) harboring genes encoding metallo-β-lactamase may be spreading in hospitals throughout Europe and Asia. *E. xiangfangensis* CC200 (ST105 and ST200) harboring *bla*_VIM-1_ has been detected in Croatia and Turkey (12), and six clinical isolates of carbapenem-resistant *E. xiangfangensis* ST200 harboring *bla*_NDM-1_ were detected at a hospital in Yangon, Myanmar (11). As summarized in Table 3, clinical isolates of *E. xiangfangensis* ST200 coharboring *bla*_NDM-1_, *armA*, and *rmtC* were obtained from all five regions/states in Myanmar, indicating that these isolates are spreading among hospitals nationwide in Myanmar. In addition, *E. xiangfangensis* ST200 coharboring *bla*_NDM-1_, *armA*, *rmtC*, and *rmtE* is emerging in Yangon Region (Table 3).

**TABLE 3:** Drug resistance genes and isolation regions of 18 isolates of *E. xiangfangensis* ST200

ST200 (18 isolates)	Drug resistance gene(s)	Region(s) (no. of isolates)
11 isolates	*bla*_NDM-1_ and *rmtC* (plasmid^*a*^), *armA* (chromosome)	Yangon (7), Naypyitaw (1), Mandalay (1), Mon (1), Magway (1)
5 isolates	*bla*_NDM-1_ and *rmtC* (plasmid^*a*^), *rmtE* (plasmid), *armA* (chromosome)	Yangon (5)
2 isolates	*bla*_NDM-4_ (plasmid)	Naypyitaw (1), Mandalay (1)

a A 57-kbp plasmid harboring *bla*_NDM-1_ and *rmtC*.

It is unclear the reason why 16 *E. xiangfangensis* isolates harbored multiple (two or three) genes encoding 16S rRNA methylases (Table 3). At least two studies reported that clinical isolates of Pseudomonas aeruginosa in India (13) and of *Enterobacteriaceae* in the United Kingdom and Ireland (14) coharbored 16S rRNA methylase genes. There is no functional reason for these isolates to harbor the multiple genes because the 16S rRNA methylases target the same residue, G1405, of 16S rRNA (3). Dissemination and accumulation of these genes in some regions, and plasmids coharboring these genes with other drug resistance genes, may result in harboring of these types of multiple 16S rRNA methylase genes.

In conclusion, this study confirmed that *E. xiangfangensis* ST200, resistant to carbapenems and multiple aminoglycosides, is spreading in Myanmar and that these isolates harbored several types of drug resistance genes on different plasmids. Although the rates of these multidrug-resistant E. cloacae complex isolates in hospitals in Myanmar were not determined in this study, we have a plan to conduct surveillance to determine the rate in Myanmar. Epidemiological surveillance is required to prevent the emergence and spread of multidrug-resistant Gram-negative pathogens harboring various types of drug resistance genes in Myanmar.

## MATERIALS AND METHODS

### DNA isolation and whole-genome sequencing.

Bacterial DNA was extracted using DNeasy blood and tissue kits (Qiagen, Tokyo, Japan), and their complete genomes were sequenced using MiSeq (Illumina, San Diego, CA). The raw reads were assembled using CLC Genomics Workbench version 10.0.1 (CLC bio, Aarhus, Denmark).

### Genomic analysis.

Species of these isolates were determined using the ANI calculator (15, 16) and dDDH (17). The complete genome sequences of 3 indicated isolates of E. cloacae complex were determined using MinION (Oxford Nanopore Technologies, Oxford, United Kingdom). The raw reads were base called by Albacore v2.3.1, and trimmer adapters were base called by Porechop v0.2.3 (https://github.com/rrwick/Porechop). The long reads generated by MinION and the short reads generated by MiSeq were assembled using Unicycler (18). The sequences of drug resistance genes were determined using ResFinder 3.1 (https://cge.cbs.dtu.dk/services/ResFinder/). Multilocus sequence typing (MLST) was performed according to the instructions of the Enterobacter cloacae MLST database (https://pubmlst.org/ecloacae/) (19).

### Phylogenetic tree and genetic structure.

Phylogenetic trees were constructed using kSNP3 ver.3.1 software (20) and visualized using FigTree ver.1.4.3 (http://tree.bio.ed.ac.uk/software/figtree/). The genetic structures were compared in Easyfig (21).

### Pulsed-field gel electrophoresis and Southern hybridization.

The locations of drug resistance genes encoding NDM-type MBLs and 16S rRNA methylases were determined by pulsed-field gel electrophoresis and Southern hybridization.

### Ethics approval.

The study protocol was approved by the Ministry of Health and Sports in the Republic of the Union of Myanmar (Ethical Committee 2016), by the ethics committee of Juntendo University (number 809), and by the Biosafety Committee, Juntendo University (approval numbers BSL2/29-1). The information allowed about patients included age, gender, and sample tissues.

### Accession number(s).

The whole-genome sequences of all 31 isolates have been deposited in GenBank as accession no. DRA009282. Three plasmids isolated in this study have been deposited in GenBank as accession no. LC511996 (118,668 bp), accession no. LC511995 (119,259 bp), and accession no. LC511997 (345,906 bp).
