# Supplementary figures and images for: Emergence and Spread of Carbapenem-Resistant and Aminoglycoside-Panresistant Enterobacter cloacae Complex Isolates Coproducing NDM-Type Metallo-β-Lactamase and 16S rRNA Methylase in Myanmar
Source: mSphere. 2020 Mar 11;5(2):e00054-20. doi: 10.1128/mSphere.00054-20 (PMC7067590; doi:10.1128/mSphere.00054-20)

FIG S1

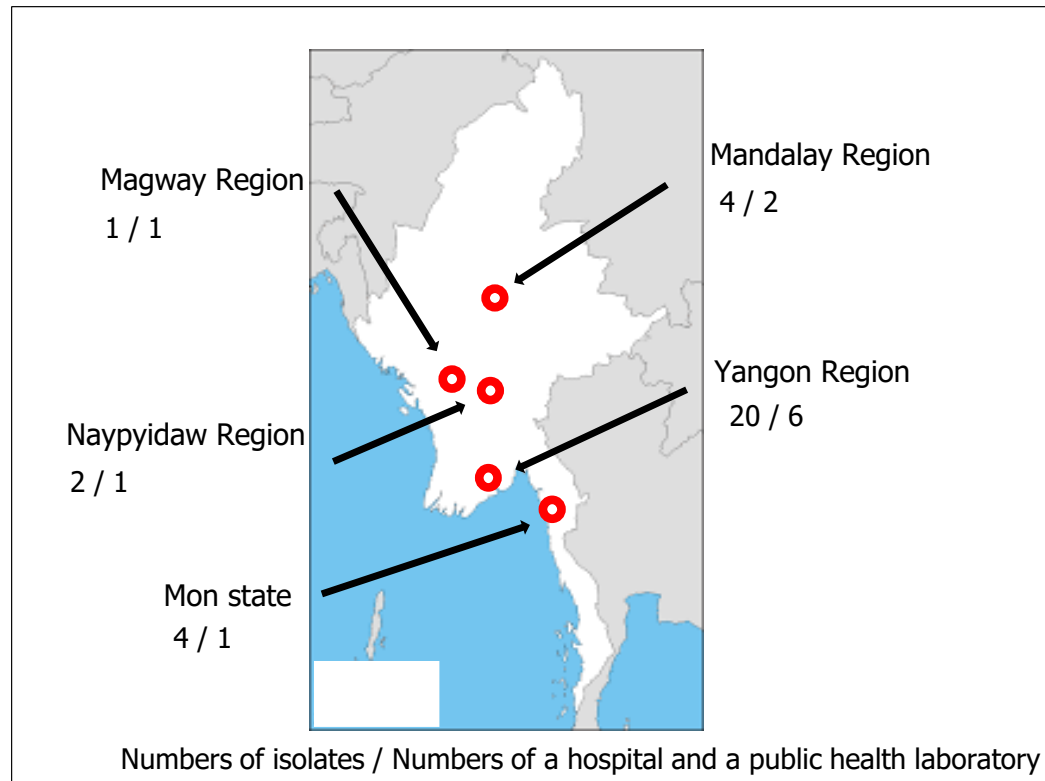

Supplement: FIG S1 [file mSphere.00054-20-sf001.pdf]

# FIG S2

(A)

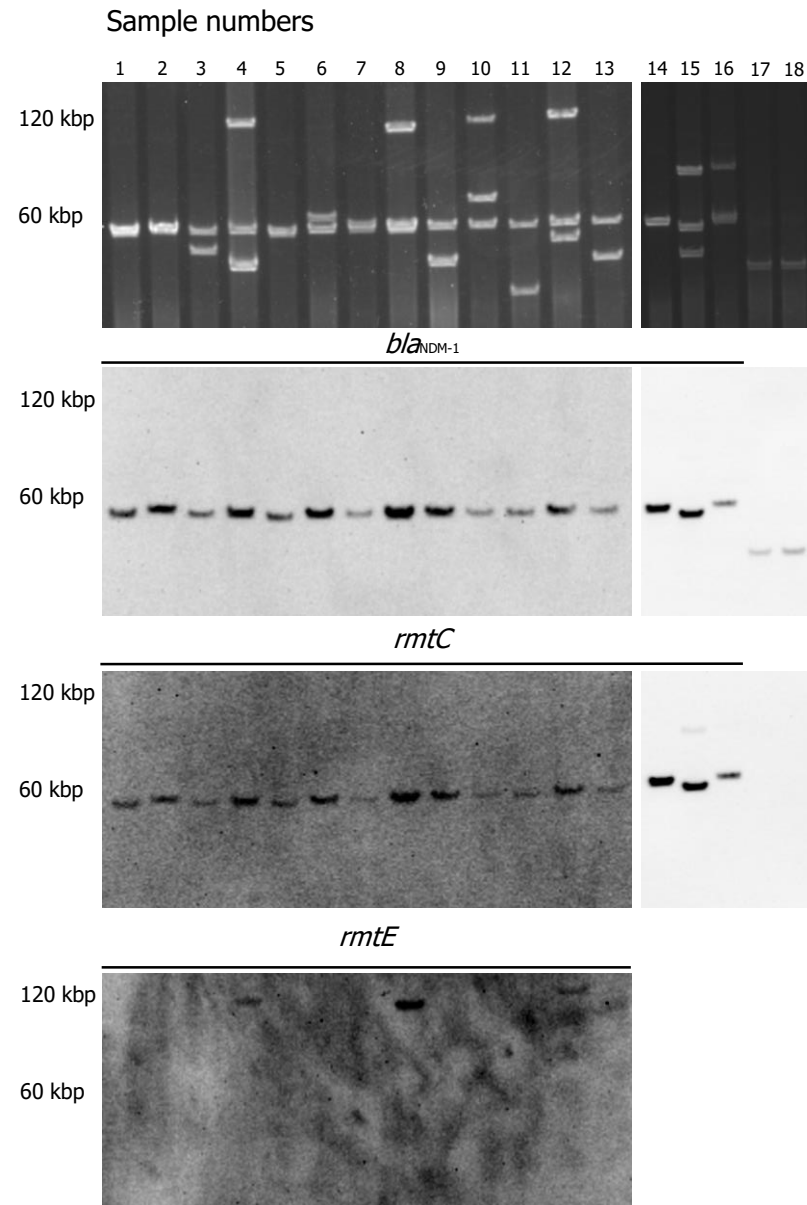

(B)

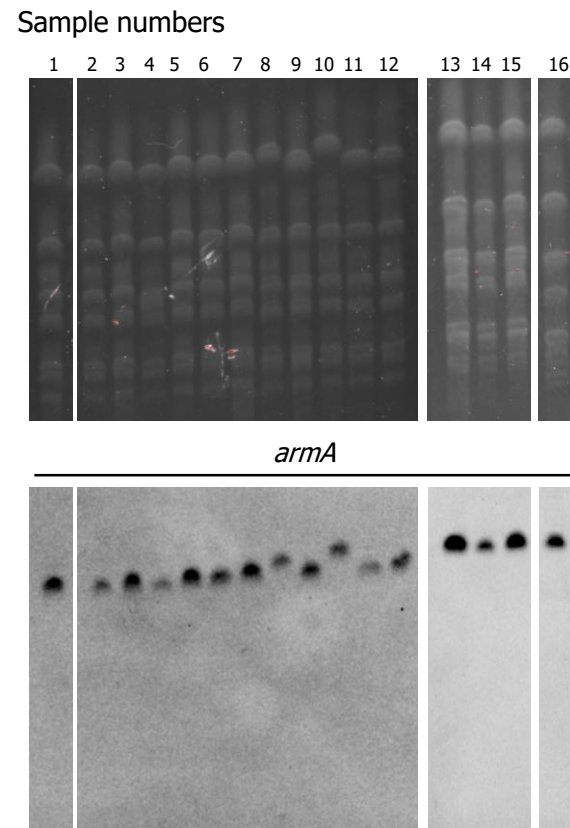

Supplement: FIG S2 [file mSphere.00054-20-sf002.pdf]

FIG S3

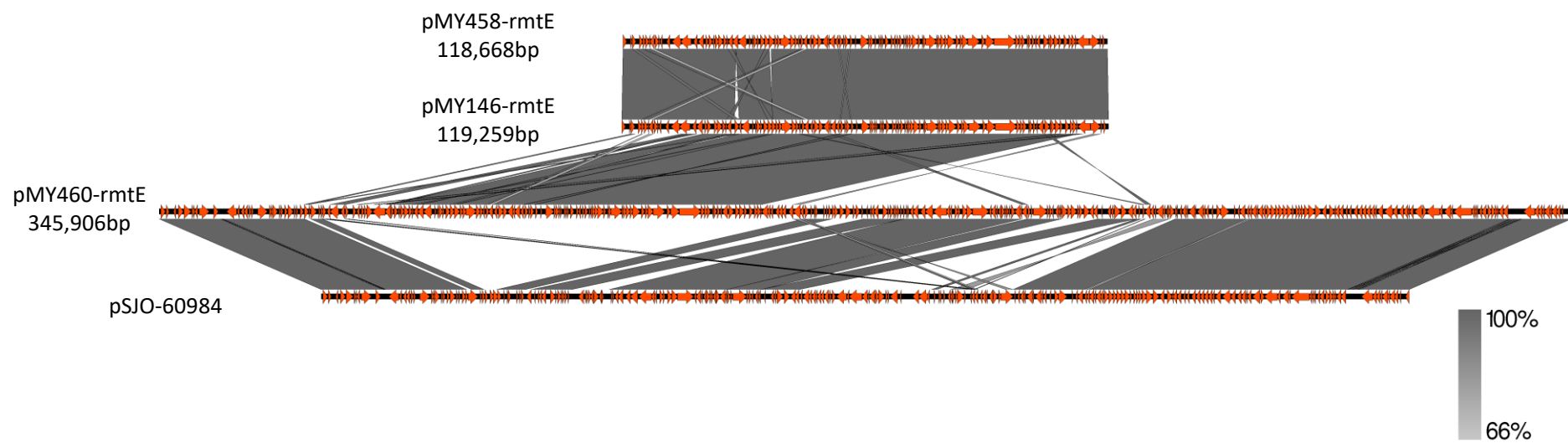

Supplement: FIG S3 [file mSphere.00054-20-sf003.pdf]
